# Special Issue “Genetics and Epigenetics of Neuromuscular Diseases”

**DOI:** 10.3390/genes14081522

**Published:** 2023-07-26

**Authors:** Fabio Coppedè

**Affiliations:** Department of Translational Research and of New Surgical and Medical Technologies, University of Pisa, 56126 Pisa, Italy; fabio.coppede@unipi.it; Tel.: +39-050-2218544

Neuromuscular disorders (NMDs) include several hereditary or acquired conditions that impair the neuromuscular system and muscle function. These include, but are not limited to, motor neuron diseases, hereditary muscular dystrophies and myopathies, peripheral neuropathies, and neuromuscular junction disorder myasthenia gravis (MG). Mutations in hundreds of genes have been linked to the inherited forms of this widely heterogeneous group of human diseases, but mounting evidence also suggests that epigenetic modifications contribute to the neuromuscular impairment observed in these conditions [1]. This Special Issue, titled “Genetics and Epigenetics of Neuromuscular Diseases”, focuses on recent advances in the genetics and epigenetics of this largely heterogeneous group of human pathologies. The collection features four original articles, one case report, and one brief report, all of which collectively contribute to enhancing our knowledge of the genetic and epigenetic factors underlying these disorders.

Pluta and co-workers [2] describe a homozygous inversion on chromosome 13 involving the γ-sarcoglycan gene, *SGCG*. This inversion was detected by short-read whole genome sequencing (WGS) in a patient suffering from limb-girdle muscular dystrophy (LGMD). LGMD refers to a large group of genetic disorders characterized by progressive muscle atrophy and weakness, predominantly affecting the voluntary muscles of the hips and shoulders. Numerous genes have been linked to LGMD that can be inherited as either an autosomal dominant or recessive disease, depending on the gene and mutation involved. Among them, homozygous or compound heterozygous mutations in one of the four sarcoglycan genes, *SGCA*, *SGCB*, *SGCG*, or *SGCD*, have been observed in patients with autosomal recessive forms of LGMD. The case study described by Pluta and co-workers involved a female patient who exhibited the first signs of progressive muscle weakness in her childhood. Subsequently, biopsy of the vastus lateralis muscle revealed the absence of γ-sarcoglycan. Initial Sanger sequencing of *SGCG* and next-generation sequencing (NGS) of a panel of genes known to cause muscular dystrophies failed to identify the causative mutation. However, the WGS approach adopted by the authors revealed a homozygous inversion on chromosome 13, resulting in a breakpoint within intron 2 of *SGCG*, leading to the absence of γ-sarcoglycan and the manifestation of disease symptoms in the affected patient. According to the authors, this is the first published case of a homozygous inversion involving *SGCG*, which was detected by short-read WGS, in a patient diagnosed with LGMD. This case also highlights the valuable contribution of this approach to the genetic diagnosis of previously unsolved cases.

In a brief report, AlMuhaizea and co-workers [3] describe two novel variants of *MEGF10* identified in two unrelated patients with congenital myopathies who were born to consanguineous parents from Saudi Arabia. Furthermore, the authors reviewed the literature and provided a list of the previously reported pathogenic variants of *MEGF10* and the associated congenital myopathies. *MEGF10* is a gene that encodes a transmembrane receptor belonging to the multiple epidermal growth factor-like domain protein family. Various *MEGF10* mutations have so far been identified in patients with neuromuscular disorders, such as minicore myopathy, LGMD, and early onset myopathy, areflexia, respiratory distress, and dysphagia (EMARDD). This report by AlMuhaizea and co-workers [3] contributes to the list of known pathogenic *MEGF10* mutations and their genotype–phenotype correlations. A case report is also included in this Special Issue, authored by Serrano-Lorenzo and coworkers [4], which focuses on metabolic myopathies. In particular, the authors describe two novel mutations in the lactate dehydrogenase (*LDHA*) gene that were identified in two unrelated Spanish patients who exhibited intolerance to anaerobic exercise and experienced episodes of rhabdomyolysis, including psoriasis-like dermatitis in one case. In addition, the authors provide an overview of the *LDHA* mutations identified thus far in individuals with metabolic myopathies and the associated clinical symptoms.

Two manuscripts in this Special Issue focus on inherited peripheral neuropathies (IPN). Jung and co-workers conducted an extensive genetic screening of Korean families with Charcot–Marie–Tooth disease (CMT) [5]. They selected 850 families with no duplication of the peripheral myelin protein 22 (*PMP22*) gene, which is the primary cause of CMT type 1A (CMT1A), and performed WGS and targeted gene sequencing to identify *PMP22* point mutations in these families. The authors identified 14 pathogenic or likely pathogenic *PMP22* mutations in 21 families, eight of which have not been reported in other countries. Although mutations in *PMP22* are the most frequent genetic cause of CMT, more than 130 different genes can also cause IPN. Among them, mutations in small heat shock protein genes, namely *HSPB1*, *HSPB8*, and *HSPB3*, cause inherited forms of CMT and other IPN. Lim and co-workers [6] conducted a comprehensive genetic screening of 758 Korean families with IPN, searching for *HSPB1*, *HSPB8*, and *HSPB3* mutations. The authors identified nine pathogenic or likely pathogenic variants of these three genes in 11 families. Both studies [5,6] provide detailed clinical and electrophysiological characterizations of the affected individuals, thus expanding the list of known *PMP22*, *HSPB1*, *HSPB8*, and *HSPB3* mutations and their corresponding genotype–phenotype correlations. 

X-chromosome inactivation (XCI) is a physiological epigenetic process that involves chromatin modification and inactivation of one of the two X chromosomes in female mammals to ensure a balanced dosage of X-linked genes between males and females. XCI occurs early during embryo development, and the decision of which X chromosome to inactivate in a particular cell is random; therefore, maternal and paternal X chromosomes have an equal probability of inactivation. A skewed XCI occurs when the inactivation of one X chromosome is favored over the other, resulting in most cells expressing maternally or paternally inherited X-linked genes, depending on which of the two X chromosomes preferentially escapes inactivation. Several autoimmune disorders are characterized by a sex bias, with a higher prevalence in women than in men. Skewed XCI patterns have often been observed in patients with autoimmune diseases, suggesting that an unbalanced dosage of certain immune-related X-linked genes may contribute to the female preponderance observed in these disorders. Myasthenia gravis is a neuromuscular disorder resulting from the production of autoantibodies targeting the neuromuscular junction proteins in skeletal muscles. Nicolì et al. [7] investigated whether a skewed XCI pattern can contribute to the female bias observed in the early onset forms of MG. The authors observed an increased skewed XCI pattern in female patients with MG compared to controls; thus, this finding expands the list of autoimmune conditions characterized by skewed XCI. 

Collectively, this Special Issue expands our understanding of the genetic variants underlying neuromuscular disorders, further elucidating their genotype–phenotype correlations. It also presents additional examples of epigenetic mechanisms that may potentially contribute to these disorders.
